# Differential expression of microRNA-206 and its target genes in preeclampsia

**DOI:** 10.1097/HJH.0000000000000656

**Published:** 2015-10-01

**Authors:** Christine Akehurst, Heather Y. Small, Liliya Sharafetdinova, Rachel Forrest, Wendy Beattie, Catriona E. Brown, Scott W. Robinson, John D. McClure, Lorraine M. Work, David M. Carty, Martin W. McBride, Dilys J. Freeman, Christian Delles

**Affiliations:** a Institute of Cardiovascular and Medical Sciences, College of Medical, Veterinary and Life Sciences, University of Glasgow, Glasgow, UK; b Kazan Federal University, Kazan, Russian Federation; ∗ Christine Akehurst and Heather Y. Small contributed equally to this work.

**Keywords:** gene expression, microRNA, preeclampsia

## Abstract

Preeclampsia is a multisystem disease that significantly contributes to maternal and foetal morbidity and mortality. In this study, we used a nonbiased microarray approach to identify novel circulating miRNAs in maternal plasma that may be associated with preeclampsia.

**Methods:**

Plasma samples were obtained at 16 and 28 weeks of gestation from 18 women who later developed preeclampsia (cases) and 18 matched women with normotensive pregnancies (controls). We studied miRNA expression profiles in plasma and subsequently confirmed miRNA and target gene expression in placenta samples. Placental samples were obtained from an independent cohort of 19 women with preeclampsia matched with 19 women with normotensive pregnancies.

**Results:**

From the microarray, we identified one miRNA that was significantly differentially expressed between cases and controls at 16 weeks of gestation and six miRNAs that were significantly differentially expressed at 28 weeks. Following qPCR validation, only one miR-206 was found to be significantly increased in 28-week samples in women who later developed preeclampsia (1.4-fold change ± 0.2). The trend for increase in miR-206 expression was mirrored within placental tissue from women with preeclampsia. In parallel, *IGF-1*, a target gene of miR-206, was also found to be downregulated (0.41 ± 0.04) in placental tissue from women with preeclampsia. miR-206 expression was also detectable in myometrium tissue and trophoblast cell lines.

**Conclusion:**

Our pilot study has identified miRNA-206 as a novel factor upregulated in preeclampsia within the maternal circulation and in placental tissue.

## Abstract 2

Supplemental Digital Content is available in the text

## INTRODUCTION

Preeclampsia is a syndrome that occurs during the second half of pregnancy in approximately 1–5% of women in the developed world [1]. Preeclampsia is broadly defined by the International Society for the Study of Hypertension in Pregnancy (ISSHP) as de-novo development of hypertension and proteinuria after the 20th week of gestation. The disease contributes to maternal and perinatal morbidity and mortality as well as conferring increased long-term risk of chronic illnesses. The only effective treatment is delivery both of the baby and the placenta; therefore, the basis of the disease is thought to be predominantly due to placental dysfunction. The development of preeclampsia is multifactorial and remains incompletely understood.

MicroRNAs (miRNAs) are small noncoding RNAs that act at a posttranscriptional level to degrade target genes recognised by complementary base pairing in the 3’ untranslated region of the mRNA. miRNAs may provide a novel strategy for better understanding of preeclampsia and its diagnosis. miRNAs have been shown to play a functional role in physiological processes important in pregnancy such as placental development, trophoblast proliferation and migration and angiogenesis [2]. miRNAs also exist in circulation within a number of body fluids. Currently, the origin of circulating miRNAs is not fully understood and therefore it is not known how their levels are altered in disease, or if they have a particular target via which they can exert a functional role. Circulating miRNAs are protected from degradation by specific proteins [3,4] within exosomes [5] or microparticles [6]. This suggests that miRNA in plasma may serve an important role requiring their integrity to be preserved. Few studies into preeclampsia have assessed levels of circulating miRNAs in the plasma and have either focused on late gestation or have lacked translation into relevant tissues [7–9].

Microarray technology allows the expression of many miRNAs to be detected simultaneously creating a patient-specific profile. In this pilot study, we have used an OpenArray microarray to complete a nonbiased screen of circulating miRNA expression in plasma samples taken from 18 women who developed preeclampsia and 18 matched women with normotensive pregnancies at 16 and 28 weeks of gestation. We hypothesized that there are detectable changes in circulating miRNA expression at the preclinical stage of preeclampsia. Our aim was to identify these miRNAs and evaluate their relevance as an early biomarker and their potential pathological role in disease.

## MATERIALS AND METHODS

### Patients

#### Proteomics in pre-eclampsia cohort

For the microarray study, plasma samples from women recruited between 2007 and 2010 as part of the prospective ‘Proteomics in Preeclampsia’ (PIP) study were used. The study protocol has been described elsewhere [10]; in brief, over 4000 women were recruited across Glasgow at their initial antenatal hospital appointment. Further samples were obtained from 180 women with two or more risk factors for preeclampsia (nulliparity, age over 35 years, BMI >30 kg/m^2^, family history of preeclampsia in mother or sister [11]), at gestational weeks 16 and 28. All women were followed until delivery, when pregnancy outcomes were obtained from labour ward databases and hospital case records. Of the women sampled at gestational weeks 16 and 28, 18 (10%) developed preeclampsia and were matched for age, BMI and parity with 18 women who had normotensive pregnancies. Plasma samples were stored at −80°C until analysis.

#### Placental and myometrium tissue cohort

Placental biopsies were collected at delivery from a second cohort, independent from the PIP study, of 38 women, 19 of whom were diagnosed with preeclampsia according to ISSHP criteria and 19 normotensive individuals matched for age, BMI and parity. Myometrium specimens were obtained from a third independent cohort wherein all women were normotensive. Myometrium biopsies were taken from the upper margin of the lower segment uterine incision during caesarean section in women who delivered at term (>37 weeks’ gestation: *n* = 3 before the onset of labour and *n* = 3 in labour) and preterm (defined as 24–36 weeks: *n* = 2 before the onset of labour). All of the women in the preterm group were delivered preterm due to preeclampsia. In the term group, caesarean section was due to breech position (*n* = 2) or prior elective caesarean section (*n* = 7). In both groups, individual characteristics were recorded at the time of tissue collection and delivery details were recorded from patient notes. Customized birth weight centiles were calculated using the Gestation NetworkCentile Calculator 5.4 (http://www.gestation.net/birthweight_centiles/centile_online.htm). All biopsies were flash frozen in liquid nitrogen and stored at −80°C until analysis.

The PIP Study and the collection of placenta and myometrium tissue were approved by the West of Scotland Research Ethics Committee and adhere to the principles of the Declaration of Helsinki. All participants gave written informed consent.

### Cell culture

BeWo cells (Cat. No. CCL-98) and JAR cells (Cat. No. HTB-144) were maintained in Ham's F12K medium and modified RPMI-1640 medium, respectively, in 5% CO_**2**_ at 37°C (all cells and media from ATCC). Both media were supplemented with foetal calf serum (10%), penicillin/streptomycin (1%), 200 mmol/l l-glutamine (1%) and Fungizone (1%). C2C12 cells were maintained in Dulbecco's modified Eagle medium (DMEM; Invitrogen, Paisley, USA) supplemented with foetal calf serum (10%), 200 mmol/l l-glutamine (1%) and penicillin/streptomycin (1%). Confluent cultures were subject to a 1 : 3 subcultivation ratio using trypsin/ PBS.

### RNA extraction

RNA extraction from plasma, cells and tissues was performed using the miRNeasy mini kit (Qiagen, Hilden, Germany) according to manufacturer's instructions. Samples were processed according to manufacturer's protocol. Each sample's total RNA concentration was determined by Nanodrop (Thermo Fisher Scientific, Paisley, UK) and total RNA was stored at −80°C.

### MicroRNA profiling by OpenArray

The OpenArray real-time PCR system (Applied Biosystems/Life Technologies, Paisley, UK) was used to screen for miRNA expression levels in plasma. Total RNA from patient plasma samples was processed according to manufacturer's instructions, using the low input modifications for blood plasma samples. Briefly, reverse transcription of each sample was carried out with two separate primer pools in parallel, using 19.15 ng of total RNA per sample per reaction. Following reverse transcription, a preamplification step with 16 cycles was carried out and the resulting products diluted 1 : 20. The diluted samples, matched and grouped, were applied to OpenArray qPCR panels in a randomized fashion, for profiling of 754 miRNAs per sample. Before calculating the Ct values for each miRNA with the supplied software (Life Technologies), the default minimum fluorescence signal threshold was adjusted to 200 and the ‘subtract baseline’ method was applied. Prior to comparing miRNA expression levels, any miRNA probes on the array panels that did not meet the quality control criteria were excluded from analysis. Ct values from the microarray were not subject to normalization, as suitable stable miRNAs could not be found. Therefore, all statistical analysis was performed on raw Ct values presented in the text. miRNAs with a Ct of 35 or above were not included in analysis.

### Reverse transcriptase PCR

Gene expression RT-PCR was performed using the Taqman Reverse Transcription Kit (Applied Biosystems) according to manufacturer's instructions with 400 ng RNA input. The reaction was run on a Multi Block System Satellite 0.2 Thermo Cooler (Thermo Fisher Scientific) on the following settings: 25°C 10 min, 48°C 30 min, 95°C 5 min. miRNA RT-PCR was performed using the Taqman miRNA Reverse Transcription Kit (Applied Biosystems) according to manufacturer's instructions with 5 ng initial RNA input. The reaction was run on a Multi Block System Satellite 0.2 Thermo Cooler (Thermo Fisher Scientific) on the following settings: 16°C, 30 min; 42°C, 30 min; 85°C, 5 min.

### Quantitative PCR

Reactions were set up using the following reagents: Taqman Universal Mastermix (Applied Biosystems), dH_**2**_O and relevant Taqman probe (Applied Biosystems). miRNA protocol was run on an ABI PRISM 7900HT PCR system at the following settings: 95°C, 10 min; followed by 40 cycles of 95°C, 15 s; 60°C, 1 min. Gene expression protocol was run on an ABI PRISM 7900HT PCR system at the following settings: 95°C, 15 min; followed by 40 cycles of 95°C, 15 s; 60°C, 1 min. miRNA results were normalized using syn-cel-miR 39 as a spiked-in reference miRNA. *PPIA*, *TOP1* and *GAPDH* were chosen as reference genes for gene expression studies [12]. In order to obtain more robust results than with a single reference gene, gene expression in placenta was normalized to the geometric mean of all three reference genes [13]. *PPIA*, *TOP1* and *GAPDH* were chosen as reference genes for gene expression studies. Ct values were analysed using the 2(-delta delta Ct) method, with dCt indicating normalization to the geometric mean of the three reference genes.

### Statistical analysis

All data are represented ± standard error of the mean (SEM). Parameters measured in patient cohorts, miRNA changes identified from the microarray and all qPCR data were analysed using a Student's *t*-test where ^∗^ *P* < 0.05, ^∗∗^ *P* < 0.01 and ^∗∗∗^ *P* < 0.001.

## RESULTS

### miRNA-206 is differentially regulated in plasma from women who later developed preeclampsia

**FIGURE 1:**
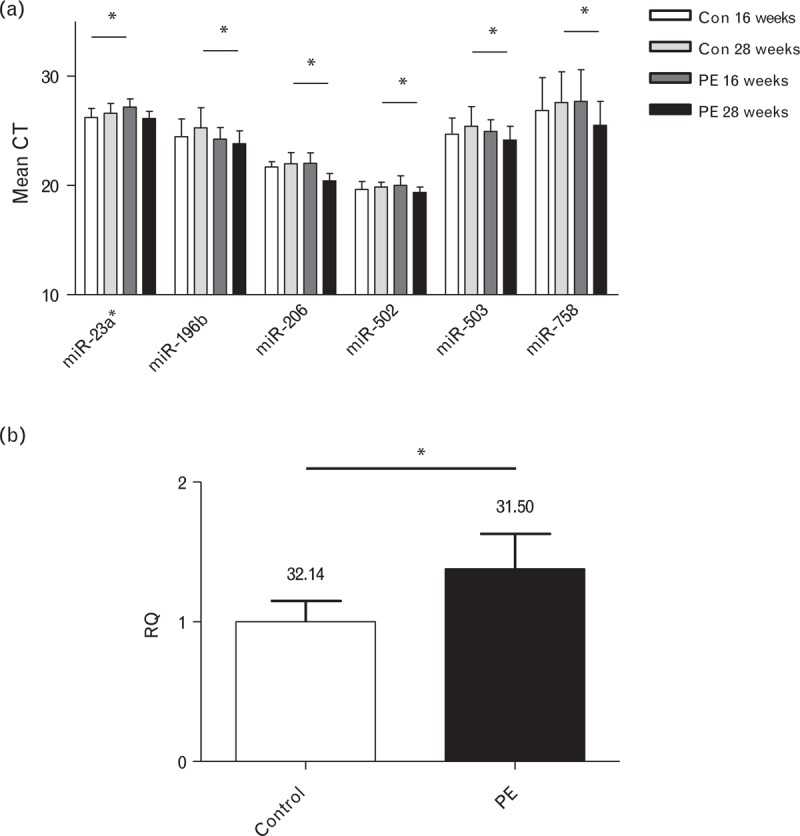
Microarray analysis shows differential miRNA expression in plasma between women who went on to develop preeclampsia and normotensive controls. (a) Six miRNAs were found to be differentially regulated between women who went on to develop preeclampsia (PE) and controls from the microarray. (b) qPCR validation shows that miR-206 expression is significantly higher in plasma samples at 28 weeks’ gestation in women who went on to develop preeclampsia, than those who did not. Raw Ct values are shown above the bar.

Plasma samples collected at 16 and 28 weeks gestation from 18 women who went on to develop preeclampsia and 18 matched normotensive women (Table 1) were subject to miRNA OpenArray analysis. Of the 754 miRNAs present on the microarray, 465 were detectable in the cohort plasma samples (Supp. Table 1, http://links.lww.com/HJH/A495). Following statistical analysis of the microarray data, six miRNAs were found to be significantly differentially expressed between the case and control groups at either time point (Fig. 1a). The only miRNA found to be differentially expressed at 16 weeks’ gestation was miR-23a^∗^, which was found to be decreased in women who went on to develop preeclampsia. The other five miRNAs identified from the OpenArray (196b-5p, 206-5p, 502-5p, 503-5p and 758-3p) all had increased expression in cases at 28 weeks of gestation only. Following these findings, the six miRNAs identified by the microarray were subject to standard qPCR validation. There were no significant differences in expression between cases and controls in miR-23a^∗^ in plasma samples taken at 16 weeks (Supp. Figure 1a, http://links.lww.com/HJH/A495). Of the differences in expression between cases and controls detected by the microarray at 28 weeks, only miR-206 was found to be significantly different between the groups in validation experiments (Fig. 1b and Supp. Figure 1b, http://links.lww.com/HJH/A495). In keeping with the microarray data, miR-206 was found to be upregulated in women who went on to develop preeclampsia (1.4-fold change ± 0.2) compared with those with normotensive pregnancies.

### miR-206 and a group of its target genes are differentially expressed in term placental tissue from women with preeclampsia

**FIGURE 2:**
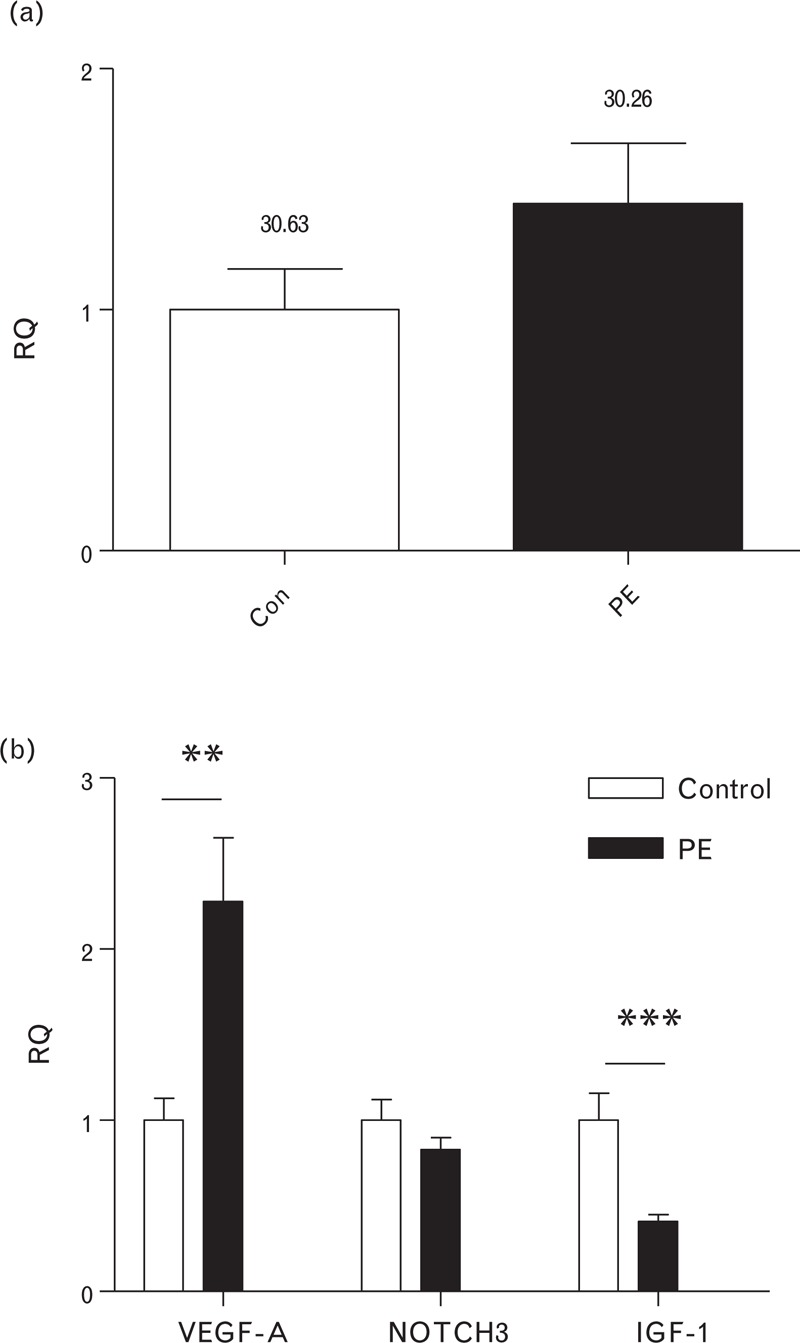
miR-206 and a group of target genes are dysregulated in placental tissue in women with preeclampsia. (a) miR-206 is higher in placental tissue of women with preeclampsia (*P* *=* 0.11). Raw Ct values are shown above the bar. (b) Targets of miR-206, VEGF-A and IGF-1 are differentially regulated in placental tissue.

Following the discovery of miR-206 dysregulation in the circulation of women who went on to develop preeclampsia, the next step was to investigate this miRNA in other tissues that have pregnancy-specific relevance. Placental samples were collected at delivery from women with preeclampsia (*n* = 19) and matched women with normotensive pregnancies (*n* = 19) (Table 2). The higher miR-206 expression seen in plasma from cases is a trend, which is mirrored in the placental tissue (Fig. 2a). Although the magnitude of increase in miR-206 in placenta (1.44-fold change ± 0.25) compared with the control group is similar to that seen for plasma, it did not reach statistical significance (*P* = 0.11) (Fig. 2a).

Interrogation of the miRNA databases TARGETSCAN-VERT [14] and miRbase [15] revealed that miR-206 targets a number of relevant genes known to be dysregulated in preeclampsia. To investigate whether expression of genes targeted by miR-206 differed in preeclampsia, the expression of vascular endothelial growth factor (*VEGF*), insulin-like growth factor 1 (*IGF-1*) and neurogenic locus notch homolog 3 (*NOTCH-3)* was also measured in the same placental tissue (Fig. 2b). There was no significant difference in *NOTCH-3* expression between placental tissue from women with preeclampsia and controls. However, two genes regulated by miR-206 were found to be differentially expressed in placental tissue. There was a significant upregulation of *VEGF* in placental tissue from women with preeclampsia (2.28-fold change ± 0.45). *IGF-1* was found to be significantly downregulated in placental tissue from cases (0.41-fold change ± 0.04).

Further investigation of miR-206 in pregnancy related tissues showed that miR-206 was detectable in both preterm women with re-eclampsia (*n* = 2) and term myometrium (*n* = 9) from normotensive women (Supp. Figure 2a, http://links.lww.com/HJH/A495). miR-206 expression was also detected in two trophoblast cell lines, BeWo and JAR (Supp. Figure 2b, http://links.lww.com/HJH/A495).

## DISCUSSION

In this pilot study, we aimed to use a nonbiased microarray approach to identify novel circulating miRNAs that are associated with preeclampsia. The microarray data showed that, amongst others, miR-206 expression was significantly increased at 28 weeks of gestation in plasma from women who later developed preeclampsia compared with those who did not. This difference in miR-206 expression is robust following qPCR validation. Furthermore, we have been able to translate this difference in circulation to biologically relevant cells and tissues. miR-206 expression is also upregulated in placental tissue from an independent cohort of women with preeclampsia. We consider this to be a major strength of this study. There was also a significant downregulation of a clinically relevant target gene, *IGF-1*, and an upregulation of another target gene *VEGF*. In addition, miR-206 was detectable in the myometrium and two human trophoblast cell lines confirming its presence in relevant tissues and supporting a potentially pathological role.

In this study, we were able to validate the change in expression in one of five miRNAs found to be differentially regulated at 28 weeks from the microarray, miR-206. Perhaps in a larger cohort, we would have had more power to identify these changes at this validation stage. The change in miR-23a^∗^ identified in the microarray at 16 weeks was not validated by qPCR. This is perhaps not surprising, as another study by Luque *et al.* [7], which looked as early as the first trimester of pregnancy, did not see any significant changes in expression either. This suggests that circulating miRNAs may not play a role in the pathogenesis of preeclampsia until later in pregnancy.

miR-206 is predominantly expressed in skeletal muscle and is part of the ‘myomiR’ family of miRNAs known to function in myogenesis and striated muscle growth and repair [16]. It is widely considered that miR-206 is exclusively expressed in skeletal muscle; however, our findings show that this is not the case. miR-206 is expressed from an intergenic region of chromosome 6 as a bicistronic transcript that also includes another myomiR, miR-133b. As a myomiR, accordingly, miR-206 has been found to be dysregulated in diseases wherein muscular dystrophy is a central mechanism [17]. However, miR-206 has also been reported to be mechanistically involved in cancer progression [18], neurological disease [19] and myocardial infarction [20]. This is the first study to define a role for miR-206 in pregnancy or preeclampsia. With respect to preeclampsia, the genomic landscape in which miR-206 is found is of interest. Downstream of miR-206 is the interleukin 17a (*IL17a*) gene that expresses IL-17, a potent cytokine mediating pro-inflammatory responses [21]. T-helper cells that produce IL-17 (TH17) have been shown to be increased in the circulation of women with preeclampsia [22]. In the same study, the cytokine itself is also found to be increased in the circulation of a rodent model of preeclampsia [22]. Whether the transcription of IL-17 and miR-206 are related is speculation but warrant further investigation.

Our findings show that miR-206 expression was observed to be higher in both the circulation and the placenta and is therefore a promising candidate for further study. Notably, it has experimentally validated interactions with a number of genes known to be directly involved in the pathology of preeclampsia, particularly at the placenta: *VEGF* [23,24], *NOTCH-3* [25,26], *IGF-1* [20] and hypoxia inducible factor 1 α (*HIF-1α*) [27]. In our study, we have shown that VEGF mRNA expression is upregulated in placental tissue from women with preeclampsia. This has been shown in other studies [28] and is thought to be a physiological response to hypoxia in the placenta. miRNAs are ‘fine-tuners’ of gene expression having small effects on large numbers of genes. miR-206 may therefore be involved in the regulation of *VEGF* expression in response to hypoxia.

Placental *IGF-1* mRNA expression was downregulated in placental tissue in preeclampsia in the present study. miR-206 has been previously been shown to target and downregulate *IGF-1* expression in other tissues, specifically rat myoblasts [20]. It is well established that maternal *IGF-1* has important growth effects on the foetus [29] and myometrial vasculature [30], which are relevant processes to the cause of preeclampsia. Paradoxically, the only study that has looked at IGF-1 protein levels in the circulation showed an increased level of *IGF-1* in the first and second trimester of pregnancy in preeclampsia [31]. It is not surprising to see conflicting results between the placenta and maternal circulation, as these are both governed by different processes. Tissue gene expression can be upregulated in order to exert local effects that are not excreted into the vasculature. Similarly, the maternal circulation is affected by shedding from all the major organs in the body as well as circulating vesicles such as exosomes and microparticles. The similar degree of upregulation of maternal plasma and placental miR-206 observed in the present study could suggest that maternal plasma miR-206 levels may originate from the placenta.

One of the strengths of our study is the screening of samples from two gestational time points, 16 and 28 weeks, both before the clinical signs of preeclampsia were apparent, whereas other studies using large-scale approaches such as microarray or next generation sequencing have only looked at one gestational timepoint [32–35]. Our study is also unique in that changes detected in the circulation by microarray have been corroborated in placental tissue from an independent cohort. Previous studies have looked at circulating miRNAs or tissue miRNAs exclusively without an overlap between the two. Circulating miRNAs have been measured in umbilical cord plasma [36], although other screens have been carried out in tissues such as the placenta [37] and trophoblasts [38].

It is unknown from our work whether the increase seen in miR-206 is a cause or effect of preeclampsia. Therefore, it can only be considered as a starting point in order to further investigate novel mechanisms of the disease. As the changes identified in our study were only seen at 28 weeks of gestation and not at an earlier time point, miR-206 upregulation may be a response to the established placental hypoxia characteristic of preeclampsia. We hypothesize that the hypoxic response leads to a compensatory increase in *VEGF* causing a parallel increase in its negative regulator, miR-206. This then results in changes in expression of other placental genes regulated by miR-206 such as *IGF-1*. Notably, placental expression of miR-206 was upregulated to a similar degree but did not reach statistical significance. We suggest that there are two reasons for this within our study. Firstly, the placenta is a diverse tissue formed by the cooperation of many different cell types. Secondly, our placental samples were representative of the entire tissue and not specific regions. It has previously been shown that miRNA levels can vary according to the region of placenta that is sampled [39]. Therefore, significant changes in miR-206 expression in one area could have been masked by nonsignificant changes in the other. A future improvement on our study is to allow for this diversity and analyse expression profiles in further dissected parts of the tissue in order for us to make a more specific statement about where such changes occur. Further to this, miRNAs are fine-tuners of gene expression; thus, changes in their expression are accordingly rapid and transient within a tissue. It may be difficult to capture miRNA upregulation at any given point in time within a tissue for such reasons, making it difficult to collect expression data without an intrinsic element of variability. Placental samples used were also not matched for gestational age. This is a generic limitation of such studies, as tissues and bloods cannot be matched for different gestational time points. Further analysis of our expression data in placental tissue shows no significant correlation between expression and gestational day of delivery (Supp. Figure 3, http://links.lww.com/HJH/A495).

In conclusion, earlier diagnosis and better understanding of the disease of preeclampsia is at the forefront of preeclampsia research. Our study has identified miR-206 as a novel differentially regulated miRNA in preeclampsia that may give information regarding the pathology of the disease. As this is a pilot study, a large independent cohort is needed to determine whether the small increase in miR-206 expression seen is reproducible and, if so, provides further insight into the complex disease of preeclampsia.

## ACKNOWLEDGEMENTS

The study was funded by grants from the European Union (EU-MASCARA; project reference 278249) and the Scottish Government's Chief Scientist Office (reference ETM/196). H.Y.S is funded by a British Heart Foundation Student Fellowship. L.S. is funded by a grant from the Russian Ministry of Education and a grant from the Boehringer Ingelheim Fonds, and the work was according to the Russian Government Program of Competitive Growth of Kazan Federal University. R.F. is the recipient of an MRC studentship.

### Conflicts of interest

There are no conflicts of interest.

## Figures and Tables

**TABLE 1:** Characteristics of women in the microarray study cohort

	Cases (*n* = 18)	Controls (*n* = 18)	*P*
Age (years)	31 ± 5.3	31 ± 5.4	NS
BMI (kg/m^2^)	29.9 ± 5.3	29.9 ± 4.4	NS
Smoker (*n*)	1 (5.5%)	3 (16.6%)	NS
Nulliparous	16 (88.8%)	15 (83.3%)	NS
Previous preeclampsia	0	0	NS
Family history (mother or sister) of preeclampsia	6 (33.3%)	1 (5.5%)	0.09
SBP 16 weeks gestation (mmHg)	126 ± 10	123 ± 9	NS
DBP 16 weeks gestation (mmHg)	81 ± 8	76 ± 7	0.004^**^
SBP 28 weeks gestation (mmHg)	129 ± 10	123 ± 9	0.043^*^
DBP 28 weeks gestation (mmHg)	81 ± 8	76 ± 7	0.045^*^
Caesarean section (*n*)	4 (22.2%)	3 (16.6%)	NS
Gestation at delivery (weeks)	38.9 ± 2	40.2 ± 1.7	0.046^*^
Birth weight (g)	3134 ± 421	3632 ± 685	0.013^*^

Plasma samples taken at 16 and 28 weeks gestation from 18 women who developed preeclampsia (cases) were matched for age (30.6 ± 1.3 years), BMI (29.4 ± 1 kg/m^2^) and parity (*n* *=* 16 primigravidae) to 18 women who had normotensive pregnancies (controls). Data are presented as mean ± standard deviation.

^*^ *P* < 0.05.

^**^ *P* < 0.01.

**TABLE 2:** Characteristics of the placental tissue cohort

	Cases (*n* = 19)	Controls (*n* = 19)	*P*
Age (years)	29 ± 5.4	30 ± 4.6	NS
BMI (kg/m^2^)	29.5 ± 6.8	29.4 ± 6.7	NS
Smoker (*n*)	4 (21.1%)	5 (26.3%)	NS
Nulliparous	11	8	NS
Third trimester SBP (mmHg)	148 ± 25	123 ± 19	0.001^**^
Third trimester DBP (mmHg)	93 ± 16	73 ± 9.4	0.00005^***^
Gestation at delivery (weeks)	35.9 ± 3.2	39.4 ± 1.5	0.0001^***^
Birth weight (g)	2463 ± 840	3573 ± 652	0.00006^***^

Placental samples taken at delivery from 19 women with preeclampsia (cases) were matched for age (29.7 ± 0.7 years) and BMI (29.4 ± 6 kg/m^2^) to 18 women who had normotensive pregnancies (controls). Women with preeclampsia from this cohort, on average, delivered before full term. Data are presented as mean ± standard deviation. ^*^ *P* < 0.05.

^**^ *P* < 0.01.

^***^ *P* < 0.001.

## Reviewers’ Summary Evaluations

### Reviewer 1

This study represents a preliminary step to shed light into the pathogenesis of preeclampsia and the role of microRNAs.

### Reviewer 2

Strengths: The pathophysiology of preeclampsia remains not well defined and the existence of regional differences in the etiology of the preeclampsia has been proposed. This article identified miRNA-206 as a novel factor up-regulated in preeclampsia within the maternal circulation and in placental tissue. New multi centric, collaborative studies will be necessary to understand the real importance of circulating miRNAs in maternal plasma that may be associated with preeclampsia.

Weaknesses: Small number of patients that confers to this study the characteristics of a pilot study. It will be necessary to define the probable role of the association of microRNA-206 with down-regulation of IGF-1.
